# A statistical shape analysis for the assessment of the main geometrical features of the distal femoral medullary canal

**DOI:** 10.3389/fbioe.2024.1250095

**Published:** 2024-04-10

**Authors:** Valentina Betti, Alessandra Aldieri, Luca Cristofolini

**Affiliations:** ^1^ Department of Industrial Engineering, Alma Mater Studiorum—University of Bologna, Bologna, Italy; ^2^ PolitoBIOMed Lab, Department of Mechanical and Aerospace Engineering, Politecnico di Torino, Torino, Italy

**Keywords:** statistical shape model (SSM), medullary canal, anatomical variability, shape variation, personalized orthopedic implants, principal component analysis—PCA

## Abstract

Statistical Shape Models (SSMs) are widely used in orthopedics to extract the main shape features from bone regions (e.g., femur). This study aims to develop an SSM of the femoral medullary canal, investigate its anatomical variability, and assess variations depending on canal length. The canals were isolated from 72 CT femur scans, through a threshold-based segmentation. A region of interest (ROI) was selected; sixteen segments were extracted from the ROI, ranging from 25% of the full length down to the most distal segment. An SSM was developed to identify the main modes of variation for each segment. The number of Principal Components (PCs) needed to explain at least 90% of the shape variance were three/four based on the length of the canal segment. The study examined the relationship between the identified PCs and geometric parameters like length, radius of curvature, ellipticity, mean diameter, and conicity, reporting range and percentage variation of these parameters for each segment. The SSMs provide insights into the anatomical variability of the femoral canal, emphasizing the importance of considering different segments to capture shape variations at various canal length. These findings can contribute for the design of personalized orthopedic implants involving the distal femur.

## 1 Introduction

The femoral morphology shows a high inter-subject variability due to several reasons such as genetic factors, lifestyle factors, and/or pathologies like osteoporosis (Kiebzak, 1991; Marangalou, et al., 2014; Jung, et al., 2021). As a whole, the study of the main morphological variations of the femoral district could be pivotal for diagnosing pathologies, refining surgical procedures, and for developing reliable customized implants (Rao, 2013). More in detail, the effective design of prosthetic devices directly interacting with the femur bone should take into account the main anatomical variations of this skeletal district, as optimized results in terms of implant stability and load transfer would be achieved (Bischoff, et al., 2014). Some of the most important geometric parameters reported in the literature to describe the femur are femur length, narrowest medullary diameter, neck shaft angle, and the radius of curvature of the canal (Jung et al., 2021). However, they are discrete geometrical variables, often not independent one from the other and above all unable to consider the femur shape as a whole.

Statistical Shape Models (SSMs) allow to overcome these limitations. Over the past decades, SSMs have found widespread application in characterizing population data sets’ variability and predicting new instances within that population (Barratt, et al., 2008; Baka, et al., 2011; Aldieri, et al., 2020). In the field of orthopaedics, SSMs have been employed for many applications regarding the femur, since it is one of the most implanted districts within the skeleton (Lindner, et al., 2013; Sarkalkan et al., 2014; Noussios, et al., 2019). More in detail, SSMs have been employed to automatize the segmentation of the femur from clinical images (Bryan et al., 2010); to predict missing parts from portions of the distal femur (Ramme, et al., 2011) or to predict more complex femoral shapes from incomplete or sparse data obtained through less invasive methods (e.g., DXA images) (Humbert et al., 2017); to create new virtual instances (Pascoletti, et al., 2021; La Mattina, et al., 2023); to classify subjects and identify diseases (Waarsing, et al., 2010; Aldieri, et al., 2022).

In the field of prosthetics, SSMs were used to guide the optimal design of knee implants according to varying tibial plate sizes and shapes (Westrich, et al., 1994; Fitzpatrick, et al., 2007). Although these studies were conducted on the tibia, a similar approach could also be adopted for the femur bone, so as to investigate the number of sizes required to provide the best coverage between implant and subject (Boyde and Kingsmill, 1998). More in particular, due to the increase in the number of orthopaedic devices directly implanted inside the femur (e.g., osseointegrated stems, intramedullary nails) over the years (McLaughlin, 2018; Calder, et al., 2022), a better understanding of the medullary cavity shape variations could be helpful for various purposes. Taking into account the increase in osseointegrated prostheses for transfemoral amputees, the construction of a SSM of the medullary cavity would allow to identify its main anatomical variations in a population, also in relation to the length of the residual limb. Indeed, a minimum residual length is required to place the implant; however, if the femoral canal exceeds an optimal length, a partial resection is necessitated to accommodate adequate space for the external components (Hringsdottir, 2016; OTN Implants, 2023). In order to assess how, and to what extent, the morphology of the canal varies, it is essential to explore different lengths of the femoral canal. Besides that, no study in the literature has ever investigated how and whether the various anatomical variations depend on the residual length. Furthermore, a SSM of the medullary canal alone would also prevent the outcomes from being affected by confounding factors related to the whole femoral shape (e.g., neck shafts) and allow to investigate the only parameters that are relevant for such district, such as the diameter and the radius of curvature, according to studies on similar anatomical district in terms of shape (Chantarapanich, et al., 2008; Jauch et al., 2014; Hochreiter, et al., 2022).

In this study, a Statistical Shape Modelling framework has been adopted to investigate the medullary canal anatomical variability. The aims of this study were:

• To develop an SSM of the medullary canal, in order to identify the main modes of variation in the aforementioned district;
• To assess the dependence of the main shape variations as a function of the length of the canal examined, by developing different SSMs based on different segments of the medullary canal.

## 2 Materials and methods

**FIGURE 1:**
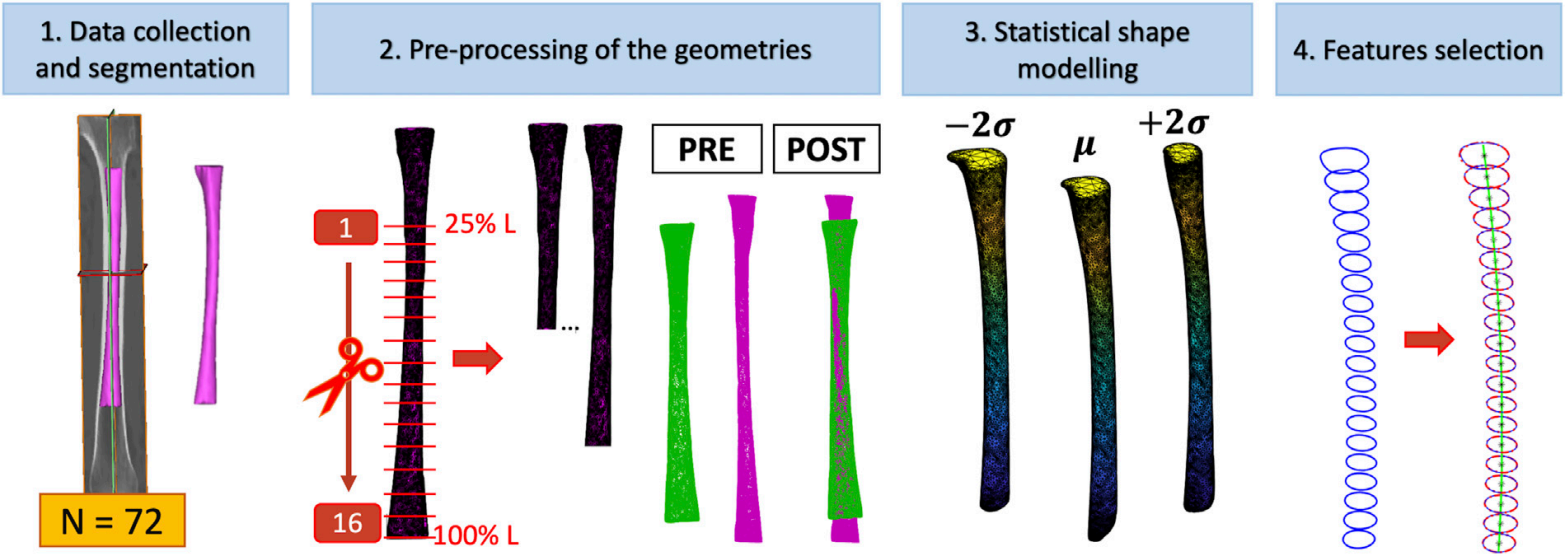
Study workflow. 1) Extraction of 3D models of the medullary from a collection of 72 CT scans of human femurs; 2) mirroring of the canals from right femurs, selection of 16 different segments of the medullary canal from the full length geometry, and alignment of the canal segments (in green) on an intermediate-size shape (in purple); 3) Statistical Shape Models (SSMs) development; 4) extraction of the most important geometric features from each shape developed.

The full pipeline which was followed in order to develop the SSMs of the medullary canal and therefore to extract the most variable geometrical parameters can be summarized in five steps (Figure 1). In the following paragraphs, each phase will be explained in detail.

### 2.1 Data collection and segmentation

The collection of a wide database of anatomical morphologies is crucial to adequately capture all the possible variations of a given bone district in a population. In the present study, 72 CT-scans of the lower limb from White donors were collected merging two different databases (Table 1):

• The scans of 22 femurs coming from the collection of *ex-vivo* specimens tested in the past by the Laboratory of Biomechanics (University of Bologna). All such previous studies have been approved by the bioethics committee of the University of Bologna.
• The scans of 50 subjects selected from the HipOp registry (Rizzoli Orthopedic Institute), including extreme cases in terms of age, weight and height, so to cover the anatomical variability as much as possible (Aldieri, et al., 2023). Informed consent was obtained from all the subjects involved in the study.

**TABLE 1:** Information about the sample used in the study. “Age” is referred to the subjects’ age at the time of the CT.

Number of subjects	Age [years]	Height [cm]	Weight [kg]
72 (43 Female, 29 Male)	67±11	165±9	73±17

For all the CT scans, the pixel size ranged between 0.41 and 0.78 mm, while the slice thickness was between 0.5–2 mm. In order to assess the minimum number of subjects required for the analysis, a convergence analysis was performed using Matlab (vers. 2022a, *MathWorks Inc*). Further details are provided in the Supplementary Material S1 (Supplementary Section S1.1).

**FIGURE 2:**
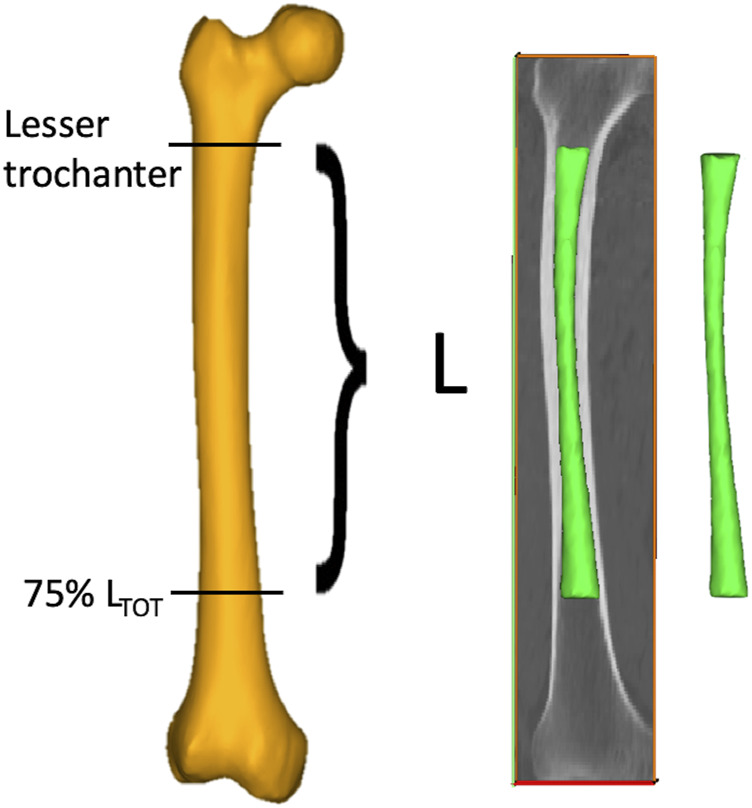
The procedure followed to obtain the medullary canals starting from the CT scans of the lower limb. A ROI (with length L) going from the lesser trochanter to the 75% of the total length of the femur (L_TOT_, measured from the tip of the greater trochanter to the intercondylar notch) was segmented from the entire femur (in yellow) to obtain the 3D models of the medullary canal (in green).

As each femur was oriented in its own CT scan reference system, the femurs were first re-aligned along their longitudinal axis (Mimics, vers. 24.0, *Materialise NV*), defined as the line connecting the lateral edge of the piriformic fossa and the intercondylar notch (Wu, 2017). A semiautomatic segmentation procedure based on thresholding [226–2999 HU] was then performed to isolate the medullary canals from the whole femur. A specific region of interest (ROI, with length L) of each subject-specific medullary canal was then identified based on the residual limb ratio definition provided by (Baum, et al., 2008) as the ratio between the residual limb length and the intact limb length. More in detail, the ROI was selected so as to consider a 75% residual limb ratio. The intact limb length was defined as the distance between the top point of the greater trochanter and the intercondylar notch. The 75% residual limb ratio was chosen in agreement with (Bell, et al., 2013; Bell, et al., 2014; Geertzen, et al., 2019), where 20%–56% residual limb ratios were associated to short residual limb subjects, while longer residual limb subjects presented a 57%–77% residual limb ratios. On top of that, the choice of the maximum 75% residual limb ratio was also considered that osseointegrated transfemoral prostheses require to include an external component, namely, the safety release system, to be set up between the internal prosthesis and the prosthetic knee (Hringsdottir, 2016; OTN Implants, 2023). This component takes up a minimum length of approximately 150 mm, thus resulting in a slightly decreased residual limb ratio, in order to align with the healthy knee. Considering that in the here considered population the largest intact limb length did not exceed 470 mm, a maximum 75% residual limb ratio was judged reasonable. The 75% residual limb ratio-based ROI was then further cropped from the tip of the lesser trochanter proximally in order to isolate the medullary canal; eventually, it was exported as a triangulated surface (Figure 2) (Ontario Health Quality, 2019; Van de Meent, 2013). As will explained in the following, smaller residual limb ratios were also considered.

### 2.2 Pre-processing of the geometries

#### 2.2.1 Mirroring

**FIGURE 3:**
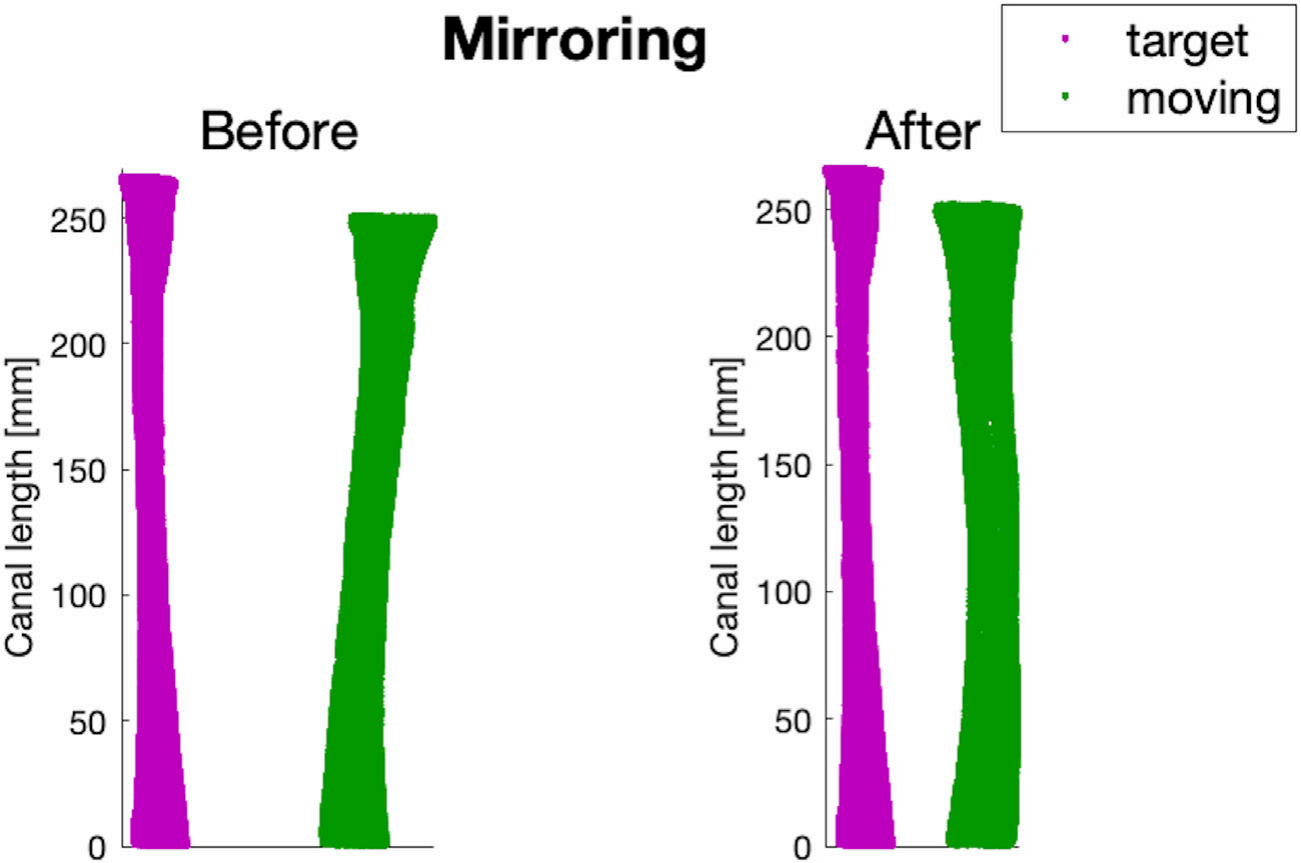
An example of before and after the mirroring step performed on a canal of a right femur from the dataset (in green), using the intermediate-sized left canal ( $M_{C}$ ) as a reference (in purple).

The canals geometries, in the form of triangulated surfaces, were imported into Matlab for additional pre-processing. Since both left and right femurs were included, a mirroring step was performed through a Procrustes analysis, using an intermediate-sized canal from a left femur $M_{C}$ as a reference (Figure 3) (Gower, 1975).

#### 2.2.2 Cutting different segments of distal femoral canal

**FIGURE 4:**
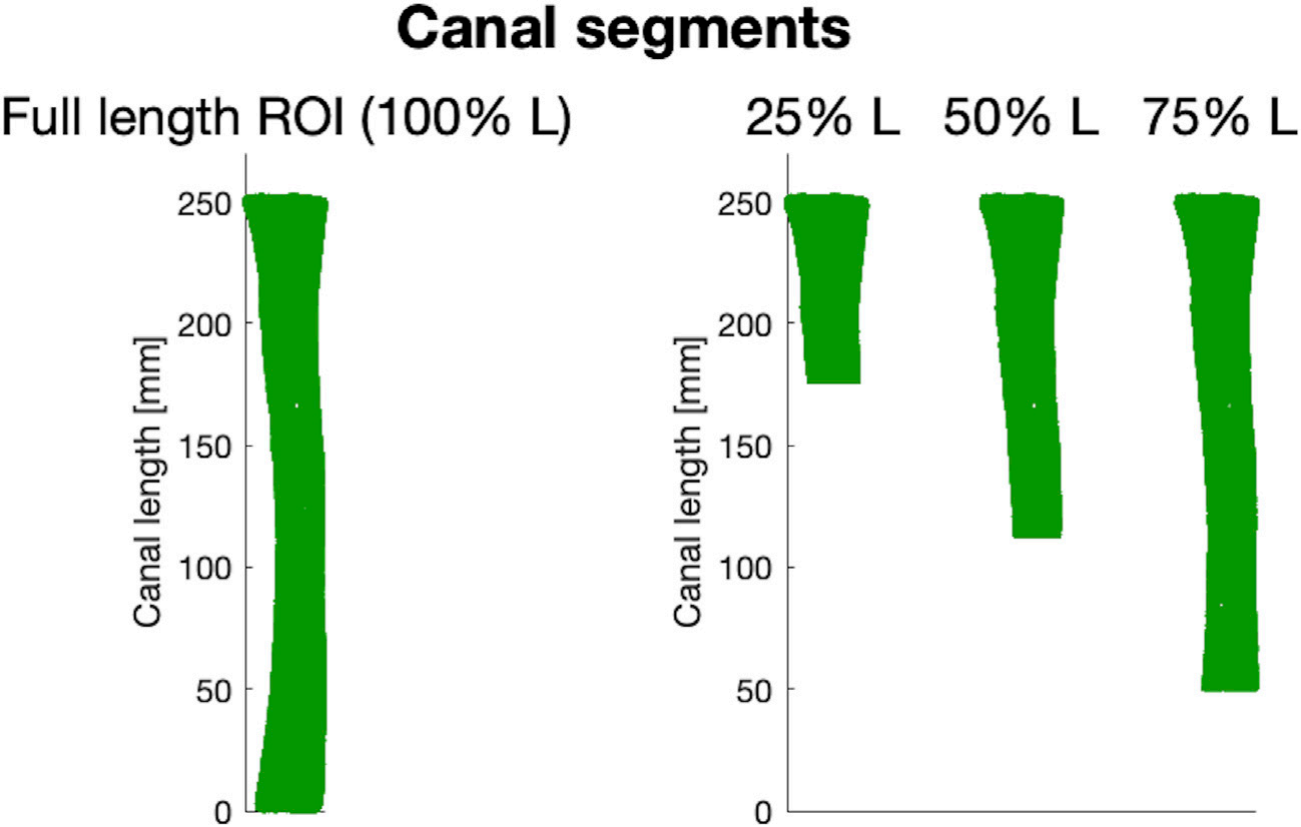
Examples of three (out of 16) canal segments considered. On the left, a 3D model of the full-length ROI from which different canal segments were obtained (25%, 50%, and 75% of the length, L, of the ROI respectively).

A major factor to be taken into account in the design of osseointegrated prosthetic devices relies on the length of the canal targeted for implantation, i.e., of the residual limb length. An amputation may in fact occur at different levels of the femur. As a consequence, patients may have different lengths of medullary canal left, where the prosthesis should be implanted. Therefore, in order to be able to consider the widest range of residual limb lengths possible, different segments of the medullary canal based on the ROI extracted as previously explained were analyzed. In this way, all the anatomical variations of the medullary canal associated to different residual limb lengths (osteotomy levels) could be captured. To this end, 16 different medullary canal segments were extracted from the full-length ROI for each of the 72 femurs, starting from a segment corresponding to 25% of the full length down (from now on, defined as 25% L) to the most distal segment of the ROI (100% L) (Figure 4). In other words, the full-length ROI was progressively cut distally, so that the medullary canal gets smaller, in order to model progressively decreasing residual limb lengths.

#### 2.2.3 Aligning all the canal segments to the same pose

**FIGURE 5:**
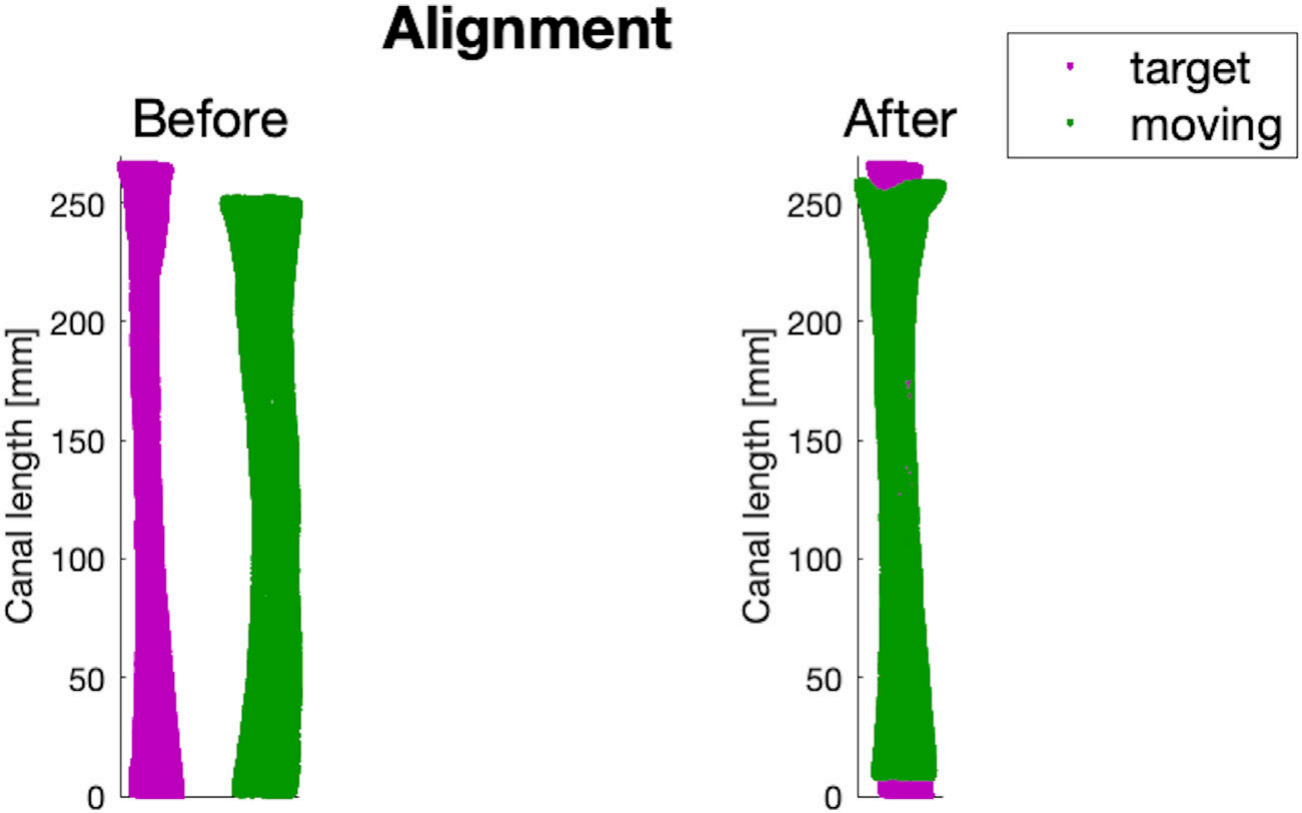
An example of before and after the alignment step performed on a canal of the dataset (in green), using the intermediate-sized canal ( $M_{C}$ ) as a reference (in purple).

In order to re-align the canals that were in different poses originally, a rigid registration was implemented using the ICP (Iterative Closest Point) algorithm. For each of the 16 different lengths of canal segments, the corresponding length of $M_{C}$ was used as the fixed target, onto which the other 71 canals were registered (Figure 5). Through a point-to-point metric, the algorithm was set to stop when the absolute difference between consecutive ICP iterations was 0.01 *mm* in translation and 0.05° in rotation. A new dataset of canals ( $M_{i,A}$ ) was then obtained.

### 2.3 Statistical Shape modelling

The construction of the Statistical Shape Models (SSMs) of the medullary canal relied on the mathematical framework proposed by Zhang, et al., 2016, implemented in the open-source Python library GIAS2 (https://gias2-shape-modelling-tutorial.readthedocs.io/en/latest/index.html). This tool allows to process a set of shapes to extract the average one and the so-called modes or principal components (PCs), i.e., the directions of highest shape variation. The workflow consists of two phases—“mesh fitting” and “shape variation analysis”—that will be described in detail below.

#### 2.3.1 Mesh fitting

In order to create a point-to-point correspondence between the meshes—and thus to obtain iso-topological geometries—the first step of the workflow consisted in an elastic registration step performed on each canal (defined as the moving object) to the intermediate-sized canal described above (defined as the target object). A radial basis function (RBF) was used to perform registration between the clouds of non-correspondent points of the target $M_{C}$ and each moving mesh $M_{i,A}$ (Broomhead, 1988). The algorithm was set to stop when the distance between each couple of these points was lower than 0.001 mm. All canals converged in 50 iterations or less. A detailed description of the process can be found in (Zhang, et al., 2014).

As a final result, a matrix $M_{i,M}$ $\in R^{N_{n}\times 3}$ (where $N_{n}$ is the number of nodes) was obtained for each of the $N_{t}$ meshes. These matrices contained the 3D coordinates of the nodes which were subsequently employed to perform the elastic registration.

#### 2.3.2 Shape variation analysis

Aiming to analyze the main modes of shape variation and the influence of the performed canal segments on the latter, an SSM was generated for each segment of canal simulated, for a total of 16 different SSMs developed.

Generally speaking, an SSM consists in the representation of the generic shape $M_{p}$ as the deformation of an average mesh $\bar{M}$ through a linear combination of principal components ${pc}_{i}$ multiplied by corresponding weights ( $w_{i}^{p}$ ) (Raudaschl and Fritscher, 2017). The average shape was obtained within an iterative process implemented in GIAS2 and which is described in (Zhang, et al., 2014). The linear combination between the principal components and their corresponding weights defines the variability model, thus providing an indication of the main directions the anatomical features vary within the cohort, and therefore of the main shape features present in the cohort. To obtain such variability model principal component analysis (PCA) was here applied to each $i^{th}$ sample in the set of meshes, thus obtaining the eigenvectors $m_{i,PC}$ and the associated eigenvalues ( $\lambda_{i}$ ). More details about the PCA can be found in (Jolliffe, 2002). In order to identify the main modes of variation of the medullary canal, the anatomical variations from the average mesh associated to the main PCs were computed as:

$$M_{i,\pm 2\sigma }=\bar{M}\pm m_{i,PC}∙2\sqrt{\lambda_{i}}$$ (1)

so as to assess the main anatomical variations in terms of shape among the population (95% confidence interval). $M_{i,\pm 2\sigma }$ are the two geometries corresponding to ±2 standard deviation from the average shape.

### 2.4 Features selection

**FIGURE 6:**
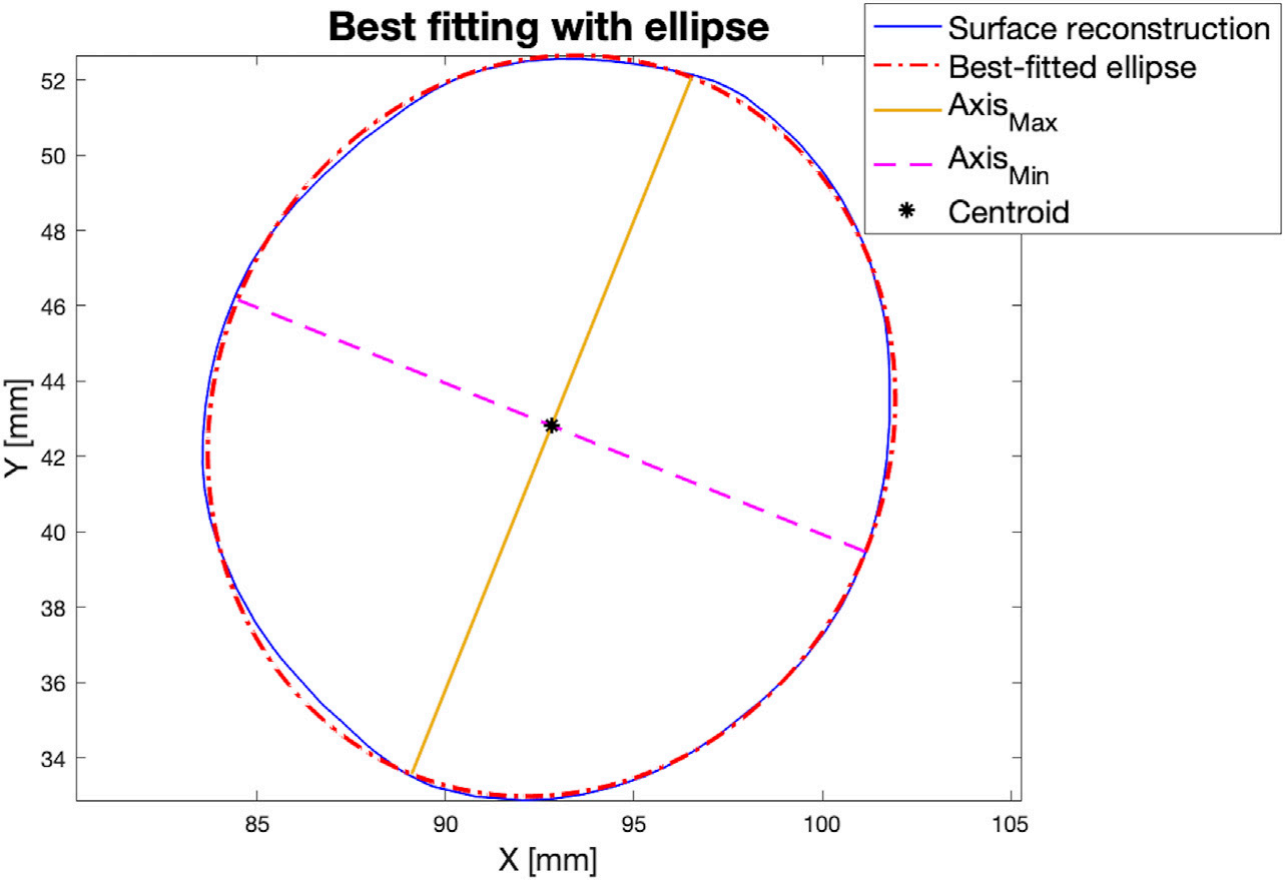
An example of a slice obtained from the reconstruction of the surface of the canal performed every 10 mm (in blue, solid line). Every surface was best-fitted with an ellipse (in red, dot-dashed line), and the maximum and minimum axes were computed (yellow, solid line and magenta, dashed line respectively). *Y*-axis represents the anterior-posterior axis (A on the top, P on the bottom, while the *x*-axis representes the medio-lateral axis (M on the right, L on the left).

For each canal segment, the shape of the canal for $\bar{M}$ and $M_{i,\pm 2\sigma }$ was reconstructed every 10 mm (with a Matlab script), thus obtaining a certain number of slices k . Every $k^{th}$ surface was then best-fitted with an ellipse, calculating the maximum and minimum axes ( ${\left({Ax}_{\max}\right)}_{k}$ and ${\left({Ax}_{\min}\right)}_{k}$ ), the centroid of the surface ${\left(x_{0},y_{0}\right)}_{k}$ , and the area ${\left(Area\right)}_{k}$ (Figure 6).

**FIGURE 7:**
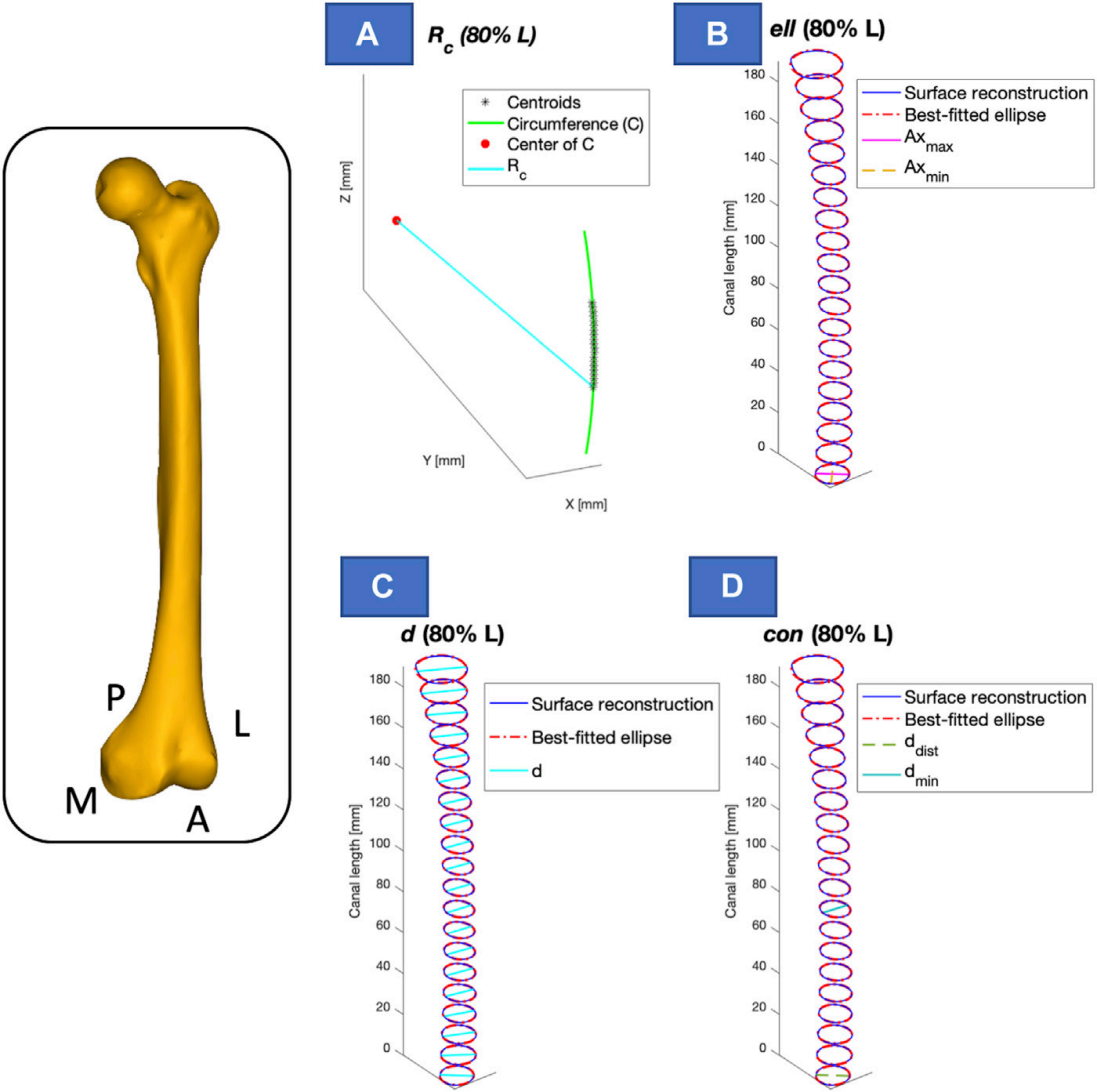
Visual representation of the calculated parameters, presented for one of the segments considered (e.g., 80% of the length - L). The longitudinal length ( $L_{a}$ ) was calculated as the canal length in the longitudinal axis. **(A)** Radius of curvature (R_c_, in cyan, solid line); **(B)** Ellipticity (ell), as the difference between axis max (in yellow, dashed line) and axis min (in magenta, solid line); **(C)** Diameters (d, in cyan, solid line) through which the mean diameter (d_avg_) was calculated; **(D)** Conicity (con), obtained as the difference between the diameter in the distal section (d_dist_, in light green, dashed line) and the diameter in the minor section (d_min_, in dark green, solid line). For a better understanding of the axis orientation, a femur is reported in yellow, indicating the anterior-posterior (A–P) and medio-lateral (M–L) axis (on the left).

The following parameters could then be calculated:

• Longitudinal length ( $L_{a}$ ): the length of the canal in the longitudinal axis.
• Radius of curvature of the canal ( $R_{c}$ , Figure 7A): the radius of curvature, $R_{c}$ , was computed by reconstructing the arc of circle passing through the centroids of the canal. The 3D coordinates of the centroids were averaged to obtain the center of gravity of the point clouds, and this value was subtracted from the respective node coordinates, thus obtaining a matrix containing the coordinates of the centroids, namely, ${\left(M_{k}\right)}_{MC}$ . In order to achieve least square fitting, singular value decomposition (*svd*) was applied on the $\left(x,y\right)$ coordinates of the matrix ${\left(M_{k}\right)}_{MC}$ to calculate the rigid rotation matrix $Q_{k}$ . The coordinates to parametrize the circle $\left(x_{curv},y_{curv}\right)$ were then derived from the multiplication between ${\left(M_{k}\right)}_{MC}$ and $Q_{k}$ . By applying a least-square fit to these coordinates $\left(x_{curv},y_{curv}\right)$ of the medullary canal, the radius of curvature $\left(R_{C}\right)$ was calculated.
• Ellipticity ( ell , Figure 7B)): the difference between ${Ax}_{\max}$ and ${Ax}_{\min}$ at the most distal section of the canal.
• Mean diameter ( $d_{avg}$ , Figure 7C): the mean of the diameters, computed from the area of each slice within the segment considered.
• Conicity ( con , Figure 7D): the difference between the diameter in the most distal section and the minimum diameter within the segment considered.

### 2.5 Metrics

The shape variations of the medullary canal were assessed reporting the variation of the five parameters ( $L_{a},R_{c},ell,d_{avg}$ , and con ) between the ±2σ deviations from the average shape ( $M_{i\pm 2\sigma }$ ) along for the main modes of variation. For a quantitative analysis, their difference divided by the average of their values were computed, thus reporting the percentage range ( $var_{L_{a}\left[\%\right]}$ ; $var_{R_{c}\left[\%\right]}$ ; $var_{ell\left[\%\right]}$ ; $var_{d_{avg}\left[\%\right]}$ ; $var_{con\left[\%\right]}$ ), and the range of values for each parameter. Any variation under 5% was considered negligible.

This analysis was repeated for each segment of canal examined, in order to evaluate the influence of each parameter on the length of the canal segment considered.

## 3 Results

In the following, the results obtained from two canal segments of all those examined are reported (40% and 100% L). These two canal segments were chosen as an example of a short and a longer segment respectively. The results obtained from all the other intermediate segments are provided in the Supplementary Material S2 (Supplementary Section S2.1).

**FIGURE 8:**
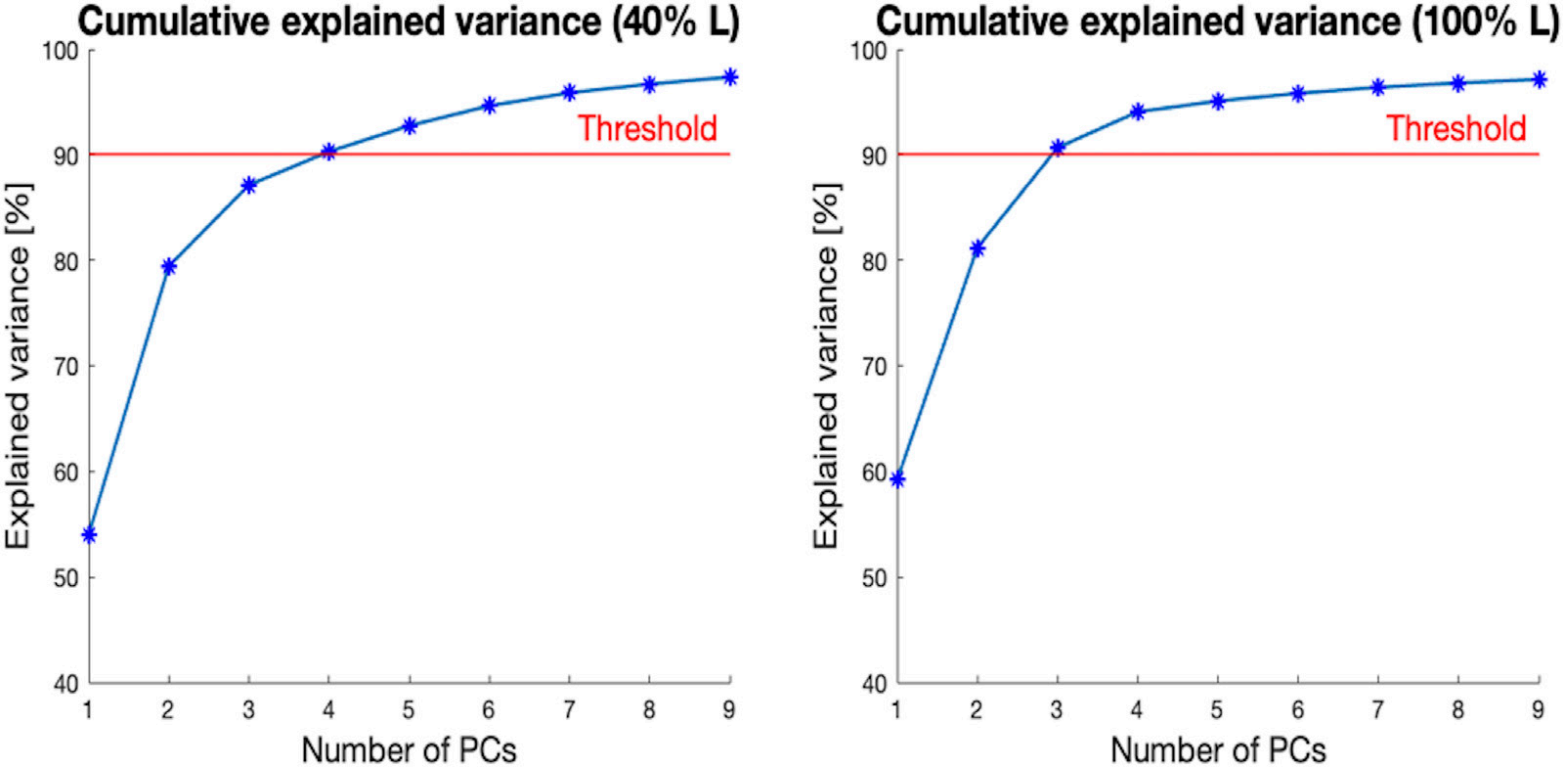
Cumulative explained variance [%] (*y*-axis) *versus* the number of principal components (PCs) for the two segments of the canal selected (40% L, on the left, and 100% L, on the right—on the *x*-axis). The red horizontal line highlights 90% of the total variance.

The number of modes required to explain at least 90% of the variance in the population ranged between three and four for the 40% L and 100% L segments (Figure 8). For the segment covering 40% of the ROI, the first four PCs accounted for 54%, 25.5%, 9%, and 3.6% respectively of the shape variance. For the 100% L segment, the first PC captured 60% of the shape variance, while the second, third and fourth PCs described 22%, 9.5%, and 3.4% respectively of the total variation.

**FIGURE 9:**
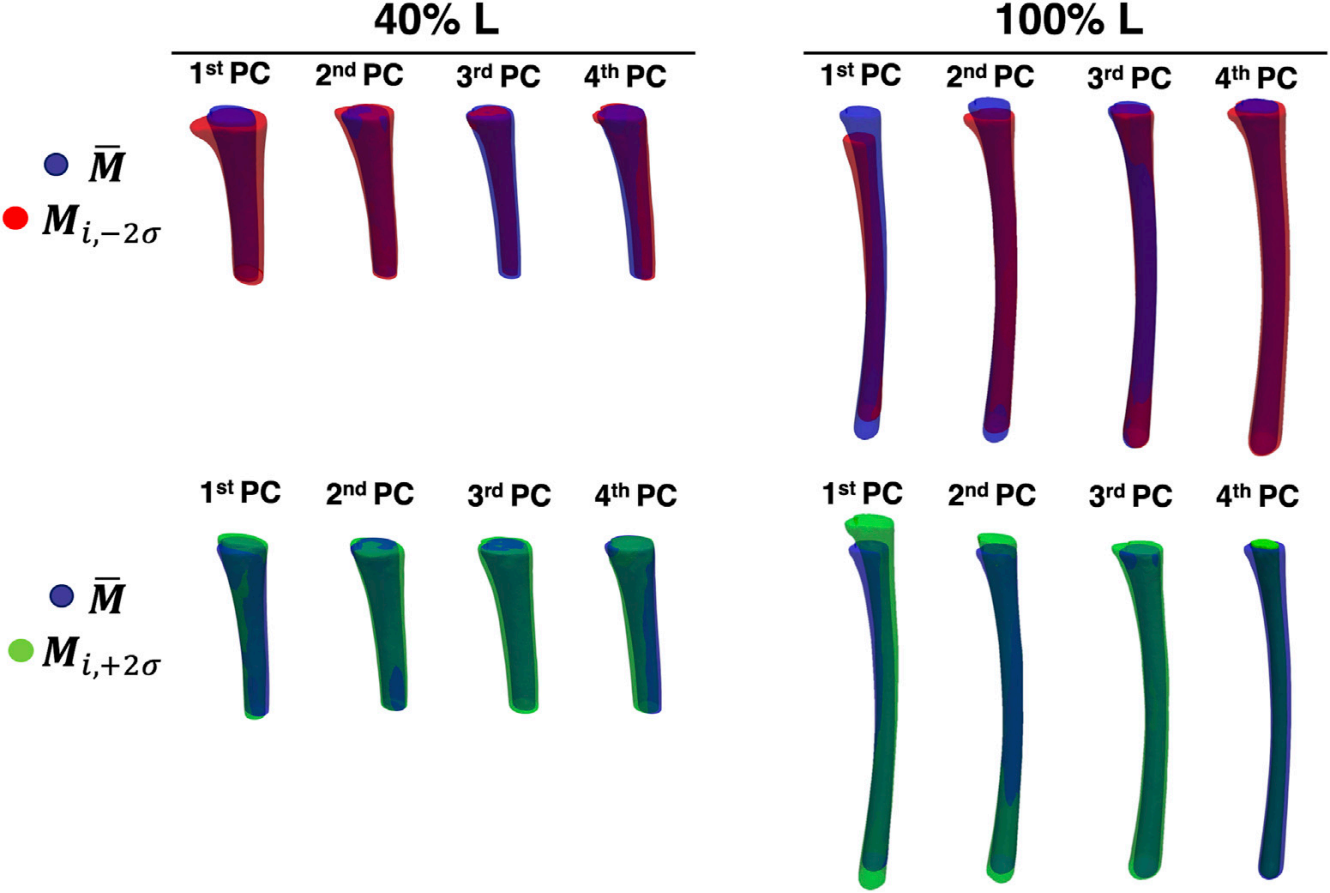
The first four PCs of the two levels of canal segment selected (40% L, on the left and 100% L, on the right). The average canal shape is reported in blue, while the shape variations along the ith PC (with a weight equal to ±2σ ) are represented in red/green.

In Figure 9, a visual representation of the main shape variations described by the four first PCs ( $M_{i,\pm 2\sigma }$ ) is provided, with reference to the average shape ( $\bar{M}$ ).

**FIGURE 10:**
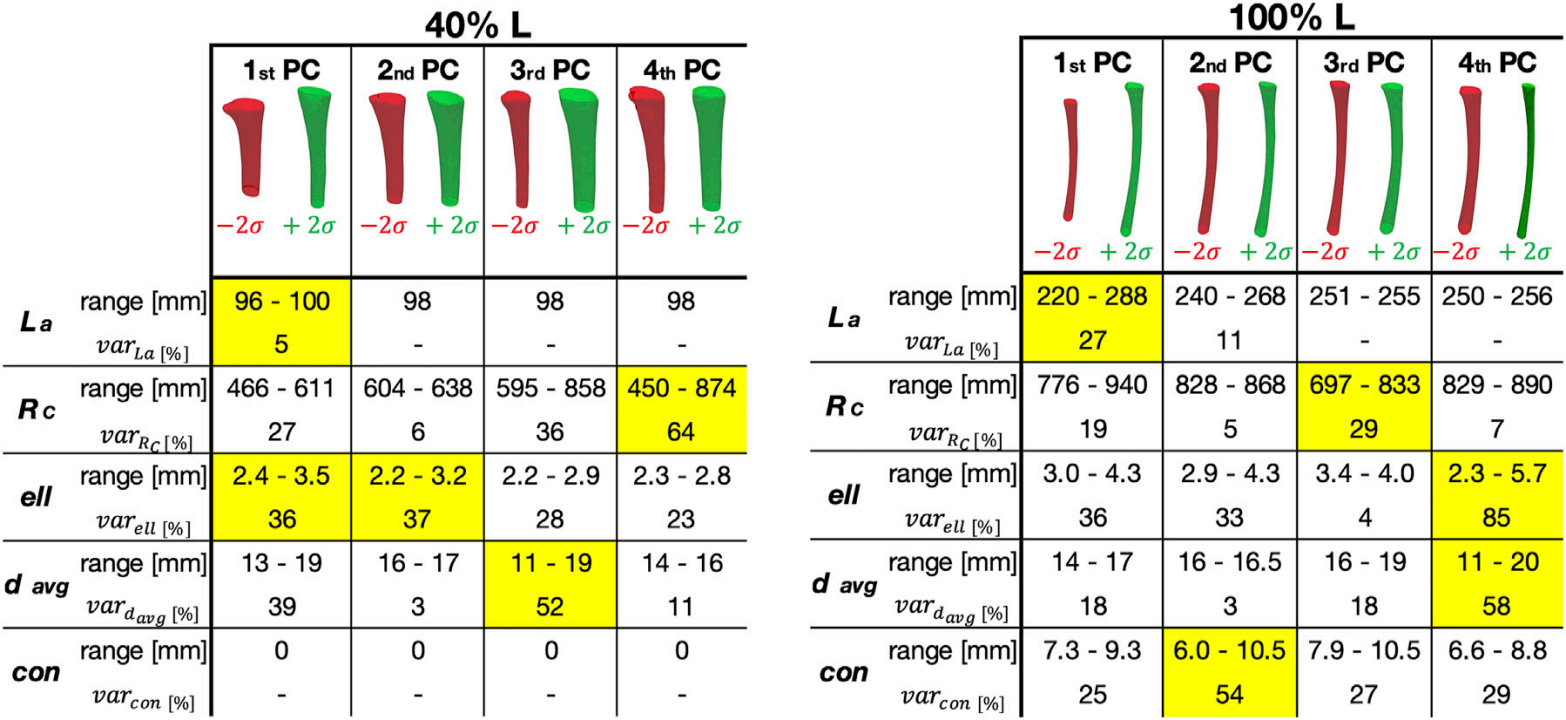
Representation of how the Principal Components (PC) correlated with the geometric parameters, for the two segments considered here (corresponding to 40% (left) and 100% (right) of the Length of the ROI—L). $L_{a}$ stands for the longitudinal length, $R_{c}$ is the radius of curvature of the canal, ell the ellipticity in the distal area, $d_{\text{avg}}$ the mean diameter, and con the conicity. Above, the main anatomical variations associated to the first four PCs. Below, for each geometric parameter, its range and the percentage variation (var) along each PC. For each geometric parameter, the column corresponding to the PC where its highest variation was observed is highlighted in yellow.

The relationship between the PCs identified as relevant, and the geometrical parameters considered is presented in Figure 10. In particular, the range and the percentage variation for each parameter (longitudinal length, radius of curvature, ellipticity, mean diameter, and conicity) is reported, for each of the first four PCs. For the 40% L canal segment, the geometric parameters with the largest variation captured by the first PC were longitudinal length ( $var_{L_{a}\left[\%\right]}=5\%$ ), and ellipticity ( $var_{ell\left[\%\right]}=36\%$ ). An equivalent high variation in terms of ellipticity was also associated to the second PC ( $var_{ell\left[\%\right]}=37\%$ ). The largest variation in terms of mean diameter ( $var_{d_{avg}\left[\%\right]}=52\%$ ), and radius of curvature ( $var_{R_{c}\left[\%\right]}=64$ %) were captured by the third and fourth PCs respectively. The influence of conicity was irrelevant for canals at such length. Nevertheless, conicity became relevant for the 100% L segment, with a variability of $var_{con\left[\%\right]}=54\%$ associated to the second PC. The first PC was here associated to the largest variations in terms of longitudinal length ( $var_{L_{a}\left[\%\right]}=27\%$ ). The radius of curvature was relevant to the third PC ( $var_{R_{c}\left[\%\right]}=29$ %), while the ellipticity and mean diameter mainly related to the fourth PC ( $var_{ell\left[\%\right]}=85\%$ ), and $var_{d_{avg}\left[\%\right]}=58\%$ ). The results for the other 14 segments of canal examined can be found in Supplementary Material S2 (Section S2.2).

**FIGURE 11:**
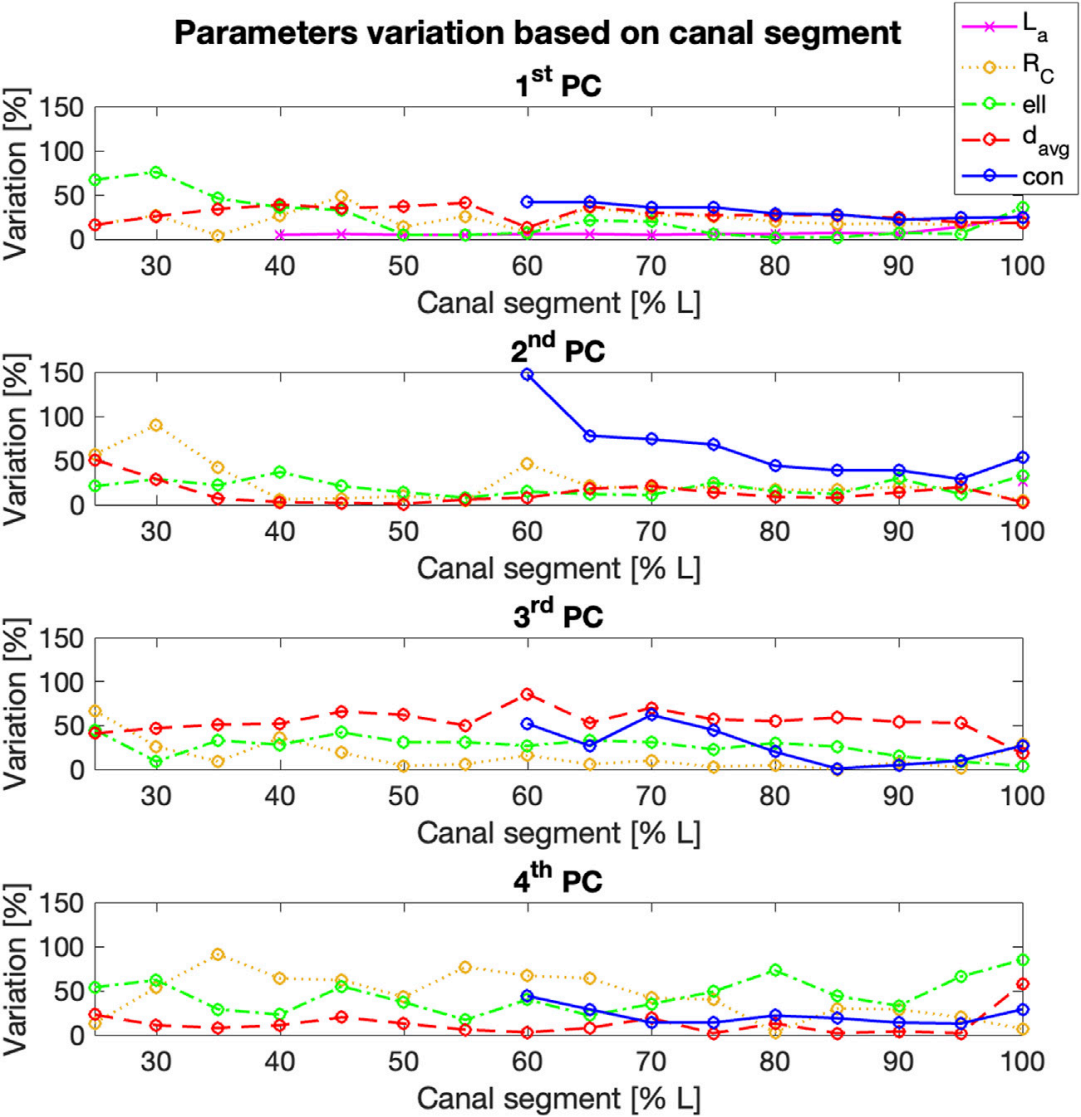
% variation of the geometric parameters examined (*y*-axis) for the first four Principal Components (PCs), for increasing length of the segment of the canal considered (*x*-axis). Longitudinal length and conicity variation was reported only from 40% L to 60% L respectively, since no significant changes were observed in the shorter canal segments.

The influence of the length of the canal segment considered on the amount of variation of each geometric parameter was also analyzed, in relation to the different PCs (Figure 11). The maximum variation in terms of radius of curvature (*R*_*C*_) could be generally observed for the fourth PC, with four exceptions (25%, 30%, 80%, and 100% L). In those cases, the highest value was associated to the second/third PCs. The highest variability was observed for the fourth PC starting from 35% L ( $var_{R_{c}\left[\%\right]}=91$ %), with values that varied between 20% and 77%. The highest variations in terms of ellipticity (*ell*) were associated mainly to the first PC from 25% L to 40% L, and to the fourth PC from 45% L to 100% L, with the highest variation for the latter ( $var_{\text{ell}\left[\%\right]}=85$ %).

The mean diameter (*d*_*avg*_) generally varied more within the third PC, with an average value around 50% and the highest values associated to 60% L ( $var_{d_{avg}\left[\%\right]}=86\%$ ). Only for 25% L and 100% L, the highest value of mean diameter was associated to another PC (second and fourth, with a variation of 67% and 58% respectively). The variability in terms of conicity (*con*) was negligible for canal segments shorter than 55% L, while it was associated to the first PC for the segments longer than 60% L. Similarly, longitudinal length variations were observed for canal segments larger than 40% L, and only associated to the first PC (and to the second PC for 100% L).

## 4 Discussion

In this study, a Statistical Shape Modelling framework was adopted in order to investigate the main geometric parameters characterizing in the medullary canal shape. In particular, starting from a 75% residual limb ratio medullary canal, different segments of the initial canal length were considered, aiming to assess if, and to what extent, the main shape variations were dependent on the length of the canal examined (i.e., amputation level). To achieve this goal, 72 CT scans were collected, 16 different segments of the canals were obtained, and a SSM was developed for each segment, aiming to investigate the shape variability present in the population at different residual limb ratios. For all the medullary canal lengths considered, the number of modes required to explain at least the 90% of the shape variation varied between three and four. As an example, when considering the level at 40% L, four components were sufficient to cover the 90% of the shape variance observed in the original database. In the analysis performed at 100% L, three components were sufficient to explain more than 90% of the variability (Figure 8). In spite of these differences, the first four components were considered for all the segments, in order to maintain consistency among the modes of variation analyzed. This number was judged reasonable, considering that nine/ten PCs for the entire femur (Taylor, et al., 2021) and 15 PCs for the mandible (Pascoletti, et al., 2021) turned out to be required to account for 90% of the variability identified in the original dataset. Considering the simple geometry of the medullary canal, it was unsurprising that a reduced number of PCs was sufficient, as the number of PCs is an indication of the complexity of a geometric shape (Skaudickas, et al., 2014).

A visual inspection of the shape variations for the first three shape modes showed large differences of the geometric parameters, also in relation to the length of the canal segment considered (Figure 9). Aiming to take advantage of the SSMs outcomes to improve the design of implants, the shapes parametrization was a crucial step to extract specific geometric parameters (Figure 10). Throughout this analysis indeed it was possible to assess the variation of these parameters and its correlation with the PCA modes. For the 40% L segment, four parameters described most of the variability of the canal: longitudinal length, ellipticity, mean diameter, and radius of curvature, were found to be associated with the first (longitudinal length and ellipticity), second (ellipticity), third (mean diameter) and fourth PC (radius of curvature). Conicity did not turn out to be significant for this segment length. The percentage of variance explained by the last three parameters was of the same order of magnitude (36/37%, 52%, and 64% of the shape variance respectively), while the length almost negligibly explained the variance (5%, Figure 8). The length became more relevant when considering longer canal segments, such as 100% L, where length variation was captured by the first PC with a higher percentage of variance considered (27%). For such canal segment, the parameters with the strongest influence were then conicity and radius of curvature, associated with the second and third PC respectively (54% and 29% of shape variance), followed by a combination of ellipticity and mean diameter both explained by the fourth PC (85% and 58% of shape variance, Figure 10). As a whole, this analysis was essential to understand that, depending on the segment of canal examined, some geometric parameters take on more importance than others.

By looking at the trend of variation of the four parameters on each canal segment analysed (Figure 11), it was possible to assess the dependence of the main geometric parameters identified on the segment of the canal considered. The quantitative measurement of this variability could be useful to define the strategies for the design of patient-matched internal prosthetic devices, thus providing insights into which anatomical parameters must be taken into account. Indeed, it has been proven how the study of the geometrical variables that define the shape of the implant can lead to the design of implants with better performances (Moradi, et al., 2021; Jia, et al., 2023), while minimizing the inefficiency and cost associated with sizing implants in the operating room (Harrysson, et al., 2007). Moreover, the identified geometric parameters might also inform the design of innovative devices that prioritize a patient-centered approach in the design process.

This work presents some limitations. Firstly, the study cohort is mainly made up of sixty-year-old subjects, meaning an upward age range. This factor may have affected the results of this study, since it has been proven that bone changes physiologically occur during aging (Kiebzak, 1991; Boyde and Kingsmill, 1998). However, the only geometric parameter which could have been influenced by the aging-process in this work is the diameter of the medullary cavity, since the total cross-sectional area of the bone becomes progressively wider with age. The addition of younger subjects would allow to evaluate if, and to what extent, this can affect the shape variations that has been observed in this study. Nevertheless, results presented in the work are primarily meant to demonstrate the potential of the presented Statistical Shape Modelling framework. For the same reason, no distinctions in terms of sex were included in this study. Since the focus was to understand the variance of the entire population, no distinctions were made either in terms of sex, age, height or other parameters. Nevertheless, the development of models that take into account such parameters could broaden the knowledge on how the geometrical variability depends on such parameters.

Another limitation relates to the lack of consideration given to bone density. In fact, while density has been considered in the so-called Statistical Shape and Intensity Models (SSIM, (Aldieri, et al., 2022)), the primary focus of this work was to evaluate the main shape features of the medullary canal, in order to make some geometric considerations useful for the design of prostheses. The bone-implant interface has undoubtedly paramount importance and it is affected by the bone quality; for this reason, additional evaluations will be necessary to include the thickness and quality of the cortical shell in the analysis.

In conclusion, this study presents the application of a Statistical Shape Modelling approach to the distal medullary canal. Several SSMs of the medullary canal were built, which allowed to include and investigate different possible residual limb ratios. This study allowed to 1) identify the main modes of shape variation associated to each canal segment, and 2) assess the dependence of these variations on the segment of the canal, i.e., on the residual limb ratio considered. The information obtained could be adopted to support the design of novel prosthetic devices able to more adequately match the anatomy of the canal with respect to the current commercial solutions.

## Data Availability

The datasets presented in this study can be found in online repositories. The names of the repository/repositories and accession number(s) can be found below: https://amsacta.unibo.it/id/eprint/7277/.
