# Supplementary material for: A statistical shape analysis for the assessment of the main geometrical features of the distal femoral medullary canal
Source: Front Bioeng Biotechnol. 2024 Apr 10;12:1250095. doi: 10.3389/fbioe.2024.1250095 (PMC11039873; doi:10.3389/fbioe.2024.1250095)
Supplement: Supplementary file 1 [file DataSheet2.PDF]

Supplementary Material #2
to the paper
“A statistical shape analysis for the assessment of the main geometrical features of the distal femoral canal”

by Valentina Betti, Alessandra Aldieri, and Luca Cristofolini

S2 Additional results

In the following, the results obtained from the 16 SSMs based on the canal segments considered are reported. The SSMs were developed by following the procedure described in the paper (Section 2.3).

S2.1 – Cumulative explained variance

The number of modes required to explain at least the 90% of the variance in the population ranged between three and four for all the segments (Fig. S2.1).

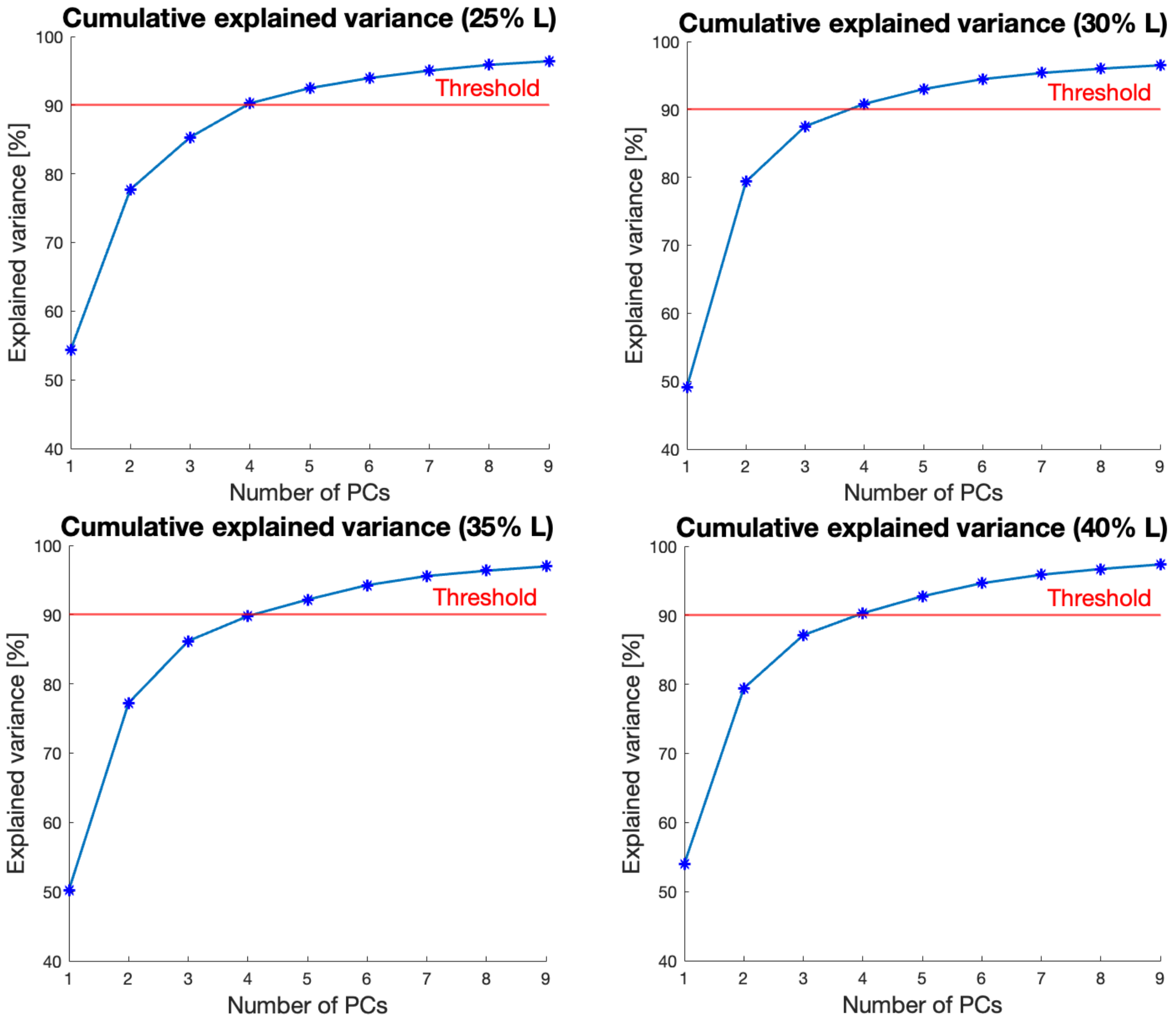

Figure S2.1: Cumulative explained variance [%] (y-axis) versus the number of principal components (PCs) for the 16 canal segments selected (x-axis). The red horizontal line highlights 90% of the total variance. ***Figure continues on next page.***

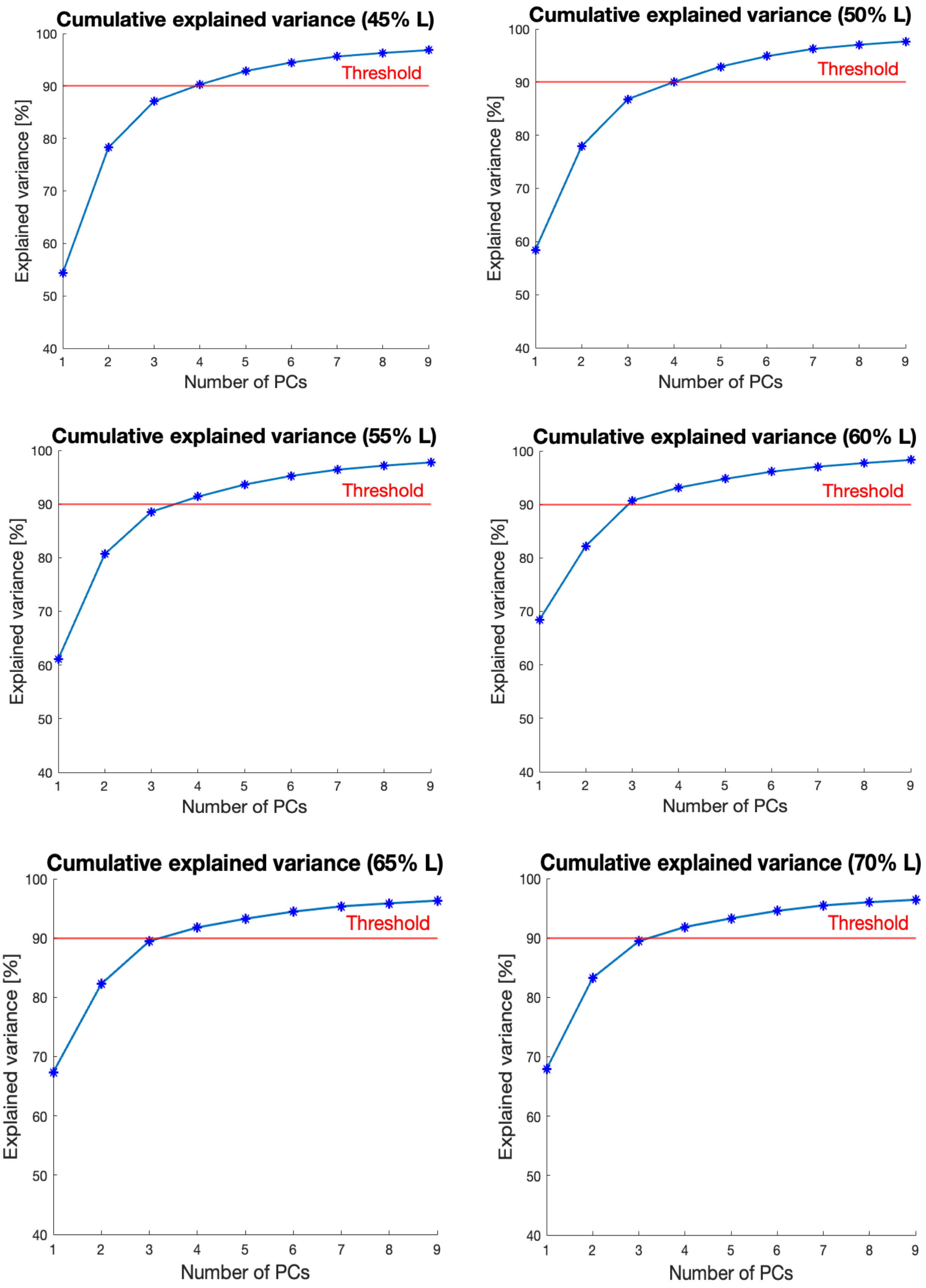

Figure S2.1: Cumulative explained variance [%] (y-axis) versus the number of principal components (PCs) for the 16 canal segments selected (x-axis). The red horizontal line highlights 90% of the total variance. ***Figure continues on next page.***

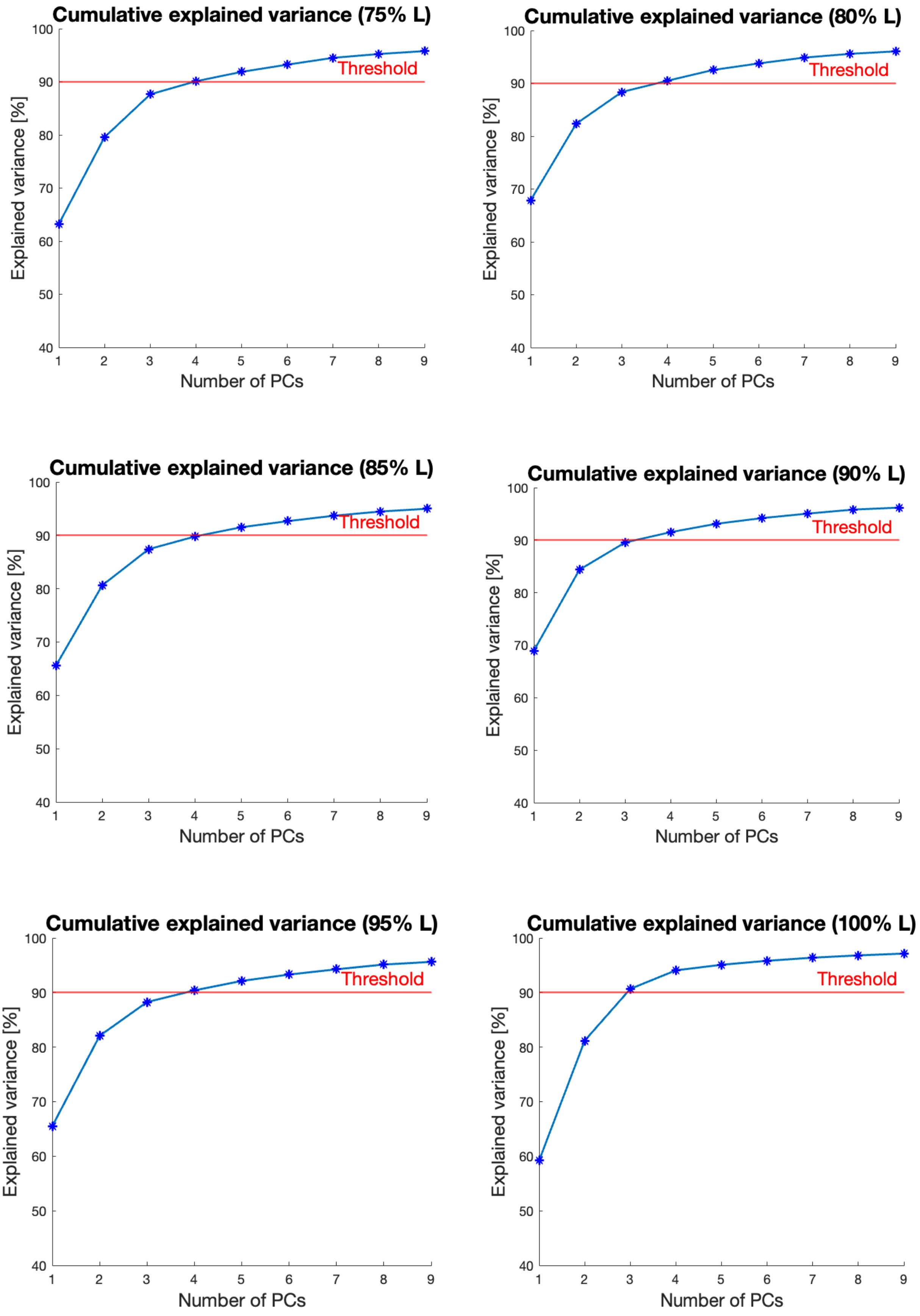

Figure S2.1: Cumulative explained variance [%] (y-axis) versus the number of principal components (PCs) for the 16 canal segments selected (x-axis). The red horizontal line highlights 90% of the total variance.

S2.2 – Relationship between principal components (PCs) and geometrical parameters

The relationship between the main PCs and the geometrical parameters considered is presented in Figure S2.2. In particular, the range and the percentage variation for each parameter (length, radius of curvature, ellipticity, mean diameter, and conicity) along each mode ($\pm 2\sigma$ variations from the average shape) is reported, for each of the first four PCs.

25% L

		1st PC	2nd PC	3rd PC	4th PC
L_a	range [mm]	60	60	60	60
	var_{La} [%]	-	-	-	-
R_c	range [mm]	548 - 641	253 - 456	600 - 1207	428 - 488
	var_{Rc} [%]	16	57	67	13
ell	range [mm]	2.0 - 4.0	2.8 - 3.4	1.8 - 2.9	2.2 - 3.9
	var_{ell} [%]	67	21	45	54
d_{avg}	range [mm]	17 - 21	12 - 21	12 - 19	14 - 18
	$var_{d_{avg}}$ [%]	16	51	41	23
con	range [mm]	0	0	0	0
	var_{con} [%]	-	-	-	-

30% L

		1st PC	2nd PC	3rd PC	4th PC
L_a	range [mm]	72 - 73	72	72	72
	var_{La} [%]	5	-	-	-
R_c	range [mm]	470 - 619	364 - 955	514 - 665	434 - 754
	var_{Rc} [%]	27	90	26	54
ell	range [mm]	1.6 - 3.6	2.6 - 3.4	2.4 - 2.6	2.1 - 4.0
	var_{ell} [%]	76	29	9	62
d_{avg}	range [mm]	17 - 22	15 - 19	13 - 20	15 - 17
	$var_{d_{avg}}$ [%]	26	29	47	11
con	range [mm]	0	0	0	0
	var_{con} [%]	-	-	-	-

35% L

		1st PC	2nd PC	3rd PC	4th PC
L_a	range [mm]	83 - 86	84	84	84
	var_{La} [%]	-	-	-	-
R_c	range [mm]	538 - 561	501 - 769	616 - 675	429 - 1153
	var_{Rc} [%]	4	42	9	91
ell	range [mm]	2.2 - 3.6	2.1 - 2.7	2.3 - 3.1	2.3 - 3.0
	var_{ell} [%]	46	22	33	29
d_{avg}	range [mm]	14 - 20	17 - 18	12 - 20	15 - 16
	$var_{d_{avg}}$ [%]	34	7	51	8
con	range [mm]	0	0	0	0
	var_{con} [%]	-	-	-	-

40% L

		1st PC	2nd PC	3rd PC	4th PC
L_a	range [mm]	96 - 100	98	98	98
	var_{La} [%]	5	-	-	-
R_c	range [mm]	466 - 611	604 - 638	595 - 858	450 - 874
	var_{Rc} [%]	27	6	36	64
ell	range [mm]	2.4 - 3.5	2.2 - 3.2	2.2 - 2.9	2.3 - 2.8
	var_{ell} [%]	36	37	28	23
d_{avg}	range [mm]	13 - 19	16 - 17	11 - 19	14 - 16
	$var_{d_{avg}}$ [%]	39	3	52	11
con	range [mm]	0	0	0	0
	var_{con} [%]	-	-	-	-

Figure S2.2: Representation of how the Principal Components (PCs) correlated with the geometric parameters, for the 16 segments considered here. For each geometric parameter, its range and the percentage variation (var) along each PC are reported. For each geometric parameter, the column corresponding to the PC where its highest variation was observed is highlighted in yellow. ***Figure continues on next page.***

45% L

		1st PC	2nd PC	3rd PC	4th PC
L_a	range [mm]	108 - 115	112	112	112
	var_{La} [%]	5	-	-	-
R_c	range [mm]	456 - 748	588 - 633	628 - 760	529 - 1001
	var_{Rc} [%]	48	7	19	62
ell	range [mm]	2.5 - 3.4	2.4 - 3.0	2.1 - 3.2	1.9 - 3.3
	var_{ell} [%]	33	21	42	55
d_{avg}	range [mm]	13 - 19	16 - 16.2	9 - 19	13 - 16
	$var_{d_{avg}}$ [%]	35	2	66	20
con	range [mm]	0	0	0	0
	var_{con} [%]	-	-	-	-

50% L

		1st PC	2nd PC	3rd PC	4th PC
L_a	range [mm]	117 - 123	120	120	120
	var_{La} [%]	5	-	-	-
R_c	range [mm]	565 - 652	618 - 686	680 - 709	552 - 850
	var_{Rc} [%]	14	10	-	43
ell	range [mm]	3.3 - 3.4	2.4 - 2.7	2.3 - 3.2	2.2 - 3.2
	var_{ell} [%]	5	14	31	37
d_{avg}	range [mm]	13 - 19	16	10 - 18	13 - 15
	$var_{d_{avg}}$ [%]	37	-	62	13
con	range [mm]	0	0	0	0
	var_{con} [%]	-	-	-	-

55% L

		1st PC	2nd PC	3rd PC	4th PC
L_a	range [mm]	128 - 137	132	132	132
	var_{La} [%]	5	-	-	-
R_c	range [mm]	588 - 767	632 - 663	662 - 702	497 - 1119
	var_{Rc} [%]	26	5	6	77
ell	range [mm]	3.3 - 3.5	2.9 - 3.2	2.3 - 3.1	2.5 - 3.0
	var_{ell} [%]	5	8	31	17
d_{avg}	range [mm]	11 - 18	16 - 17	11 - 19	14 - 15
	$var_{d_{avg}}$ [%]	41	6	50	6
con	range [mm]	0	0	0	0
	var_{con} [%]	-	-	-	-

60% L

		1st PC	2nd PC	3rd PC	4th PC
L_a	range [mm]	139 - 150	144 - 145	143 - 144	144
	var_{La} [%]	6	-	-	-
R_c	range [mm]	637 - 689	551 - 876	676 - 793	548 - 1096
	var_{Rc} [%]	8	46	16	67
ell	range [mm]	4.0 - 4.3	2.4 - 2.8	2.6 - 3.4	2.1 - 3.1
	var_{ell} [%]	7	15	27	40
d_{avg}	range [mm]	16 - 18	15 - 16	8 - 19	13
	$var_{d_{avg}}$ [%]	13	8	86	-
con	range [mm]	0.4 - 0.6	0.1 - 0.9	0.3 - 0.5	0.2 - 0.3
	var_{con} [%]	42	147	52	44

65% L

		1st PC	2nd PC	3rd PC	4th PC
L_a	range [mm]	150 - 162	155 - 156	155 - 156	155 - 156
	var_{La} [%]	6	-	-	-
R_c	range [mm]	581 - 839	593 - 731	693 - 735	536 - 1034
	var_{Rc} [%]	36	21	6	64
ell	range [mm]	2.6 - 3.4	2.4 - 2.7	2.2 - 3.1	2.5 - 3.2
	var_{ell} [%]	21	12	33	22
d_{avg}	range [mm]	12 - 17	15 - 17	11 - 18	14 - 15
	$var_{d_{avg}}$ [%]	37	18	53	8
con	range [mm]	1.0 - 1.6	0.5 - 1.1	0.8 - 1.0	0.4 - 0.5
	var_{con} [%]	42	78	27	29

70% L

		1st PC	2nd PC	3rd PC	4th PC
L_a	range [mm]	162 - 174	167 - 168	167 - 168	167 - 168
	var_{La} [%]	5	-	-	-
R_c	range [mm]	626 - 824	598 - 715	686 - 762	595 - 908
	var_{Rc} [%]	27	18	10	42
ell	range [mm]	2.7 - 3.3	2.5 - 2.8	2.2 - 3.1	2.5 - 3.6
	var_{ell} [%]	20	11	31	35
d_{avg}	range [mm]	12 - 17	15 - 18	11 - 17	13 - 15
	$var_{d_{avg}}$ [%]	30	21	70	19
con	range [mm]	1.5 - 2.2	0.8 - 1.6	0.9 - 1.7	0.6 - 0.8
	var_{con} [%]	36	74	62	14

Figure S2.2: Representation of how the Principal Components (PCs) correlated with the geometric parameters, for the 16 segments considered here. For each geometric parameter, its range and the percentage variation (var) along each PC are reported. For each geometric parameter, the column corresponding to the PC where its highest variation was observed is highlighted in yellow. ***Figure continues on next page.***

75% L

		1st PC	2nd PC	3rd PC	4th PC
La	range [mm]	174 - 186	179	179 - 180	179 - 180
	var_{La} [%]	6	-	-	-
Rc	range [mm]	625 - 815	596 - 729	709 - 732	608 - 907
	var_{Rc} [%]	26	20	-	40
ell	range [mm]	2.7 - 2.9	2.2 - 2.8	2.6 - 3.3	2.0 - 3.4
	var_{ell} [%]	6	25	46	27
d avg	range [mm]	12 - 16	15 - 17	10 - 18	14
	$var_{d_{avg}}$ [%]	27	14	57	-
con	range [mm]	1.8 - 2.6	1.3 - 2.6	1.5 - 2.4	1.4 - 1.6
	var_{con} [%]	36	68	45	14

80% L

		1st PC	2nd PC	3rd PC	4th PC
La	range [mm]	185 - 196	191	192 - 193	191 - 193
	var_{La} [%]	6	-	-	-
Rc	range [mm]	640 - 784	627 - 746	708 - 743	720 - 740
	var_{Rc} [%]	20	17	5	-
ell	range [mm]	2.9 - 3.0	2.3 - 2.7	2.5 - 3.3	2.1 - 4.6
	var_{ell} [%]	-	15	44	61
d avg	range [mm]	13 - 17	16 - 17	11 - 19	14 - 16
	$var_{d_{avg}}$ [%]	27	9	55	13
con	range [mm]	2.5 - 3.4	2.2 - 3.5	2.5 - 3.1	2.1 - 2.6
	var_{con} [%]	29	44	20	22

85% L

		1st PC	2nd PC	3rd PC	4th PC
La	range [mm]	195 - 208	203 - 204	204	203 - 204
	var_{La} [%]	7	-	-	-
Rc	range [mm]	672 - 793	645 - 766	744 - 746	656 - 890
	var_{Rc} [%]	17	17	-	30
ell	range [mm]	2.8	2.5 - 2.8	2.6 - 3.4	2.0 - 3.2
	var_{ell} [%]	-	12	48	22
d avg	range [mm]	13 - 17	16 - 17	10 - 19	14
	$var_{d_{avg}}$ [%]	27	8	59	-
con	range [mm]	3.5 - 4.7	3.4 - 5.1	3.8	3.0 - 3.7
	var_{con} [%]	28	39	-	19

90% L

		1st PC	2nd PC	3rd PC	4th PC
La	range [mm]	207 - 220	215	215 - 216	215 - 216
	var_{La} [%]	6	-	-	-
Rc	range [mm]	694 - 829	652 - 794	741 - 803	674 - 899
	var_{Rc} [%]	18	20	8	29
ell	range [mm]	2.8 - 2.9	2.1 - 2.9	2.8 - 3.3	2.2 - 3.0
	var_{ell} [%]	7	30	42	5
d avg	range [mm]	13 - 16	16 - 18	11 - 19	14
	$var_{d_{avg}}$ [%]	24	14	54	-
con	range [mm]	4.8 - 5.9	4.7 - 7.0	4.9 - 5.2	3.7 - 4.3
	var_{con} [%]	22	39	5	14

95% L

		1st PC	2nd PC	3rd PC	4th PC
La	range [mm]	216 - 250	228	227 - 228	227 - 228
	var_{La} [%]	14	-	-	-
Rc	range [mm]	720 - 849	671 - 815	786 - 804	730 - 889
	var_{Rc} [%]	16	19	-	20
ell	range [mm]	2.7 - 2.9	2.6 - 2.9	2.7 - 2.9	1.9 - 3.8
	var_{ell} [%]	6	12	66	9
d avg	range [mm]	14 - 17	15 - 18	11 - 19	15
	$var_{d_{avg}}$ [%]	19	20	53	-
con	range [mm]	5.9 - 7.5	5.7 - 7.7	6.1 - 6.7	5.0 - 5.7
	var_{con} [%]	24	29	10	13

100% L

		1st PC	2nd PC	3rd PC	4th PC
La	range [mm]	220 - 288	240 - 268	251 - 255	250 - 256
	var_{La} [%]	27	11	-	-
Rc	range [mm]	776 - 940	828 - 868	697 - 833	829 - 890
	var_{Rc} [%]	19	5	29	7
ell	range [mm]	3.0 - 4.3	2.9 - 4.3	3.4 - 4.0	2.3 - 5.7
	var_{ell} [%]	36	33	4	85
d avg	range [mm]	14 - 17	16 - 16.5	16 - 19	11 - 20
	$var_{d_{avg}}$ [%]	18	3	18	58
con	range [mm]	7.3 - 9.3	6.0 - 10.5	7.9 - 10.5	6.6 - 8.8
	var_{con} [%]	25	54	27	29

Figure S2.2: Representation of how the Principal Components (PCs) correlated with the geometric parameters, for the 16 segments considered here. For each geometric parameter, its range and the percentage variation (var) along each PC are reported. For each geometric parameter, the column corresponding to the PC where its highest variation was observed is highlighted in yellow.
