# Supplementary material for: A statistical shape analysis for the assessment of the main geometrical features of the distal femoral medullary canal
Source: Front Bioeng Biotechnol. 2024 Apr 10;12:1250095. doi: 10.3389/fbioe.2024.1250095 (PMC11039873; doi:10.3389/fbioe.2024.1250095)
Supplement: Supplementary file 2 [file DataSheet1.PDF]

Supplementary Material #1
to the paper
"A statistical shape analysis for the assessment of the main geometrical features of the distal femoral canal"

by Valentina Betti, Alessandra Aldieri, and Luca Cristofolini

S1 Convergence analysis

In order to create the Statistical Shape Model of the femoral canal, CT-scans were needed to obtain the 3D models of such anatomical district. As reported in the article, CT scans were collected from two different databases:

- CT-scans of femurs coming from a collection of *ex vivo* specimens tested in the past by the Laboratory of Biomechanics (University of Bologna, N=22). This dataset was entirely used;
- CT-scans from HipOp registry (Rizzoli Orthopedic Institute, N=200+) A subset of this dataset was selected as described below.

Since the latter is a large database, a convergence analysis was performed so as to identify the sufficient number of subjects to add in order to achieve statistically accurate results. After having obtained the 22 3D models from the first database, the femoral canals were aligned and oriented using a mean-sized left canal as described in Section 2.2 of the paper. For each of the 22 femurs, three different femoral canal segments were extracted from the ROI full length (L): 25 % L, 50% L and 100% L. Three SSMs (one for each canal segment) were then created as described in Section 2.4, and Principal Component Analysis (PCA) was performed, thus obtaining the variance in shape explained by the principal components (PCs). PCA was later repeated by including additional canals to the staring database, and the difference in the variance explained by each PC was computed each time. Canals were progressively added until this variation settled under a fixed threshold of 1%. To reach this cut-off value, four additional iterations of the models were required, including an increasing number of canals (N=50, 60, 68 and 72 respectively). Since the variation between the shape variance explained by the first three PCs between the SSMs created with 68 canals and 72 canals was under 1%, no more canals were added (Figure S1.1).

In order to verify if the shape variance described by the PCs was similar, the shapes obtained by varying the average mesh of $+2\sigma$ deviations along the first mode were compared for the 50 and 72 femurs-based SSMs (RMSE = 0.01, Figure S1.2).

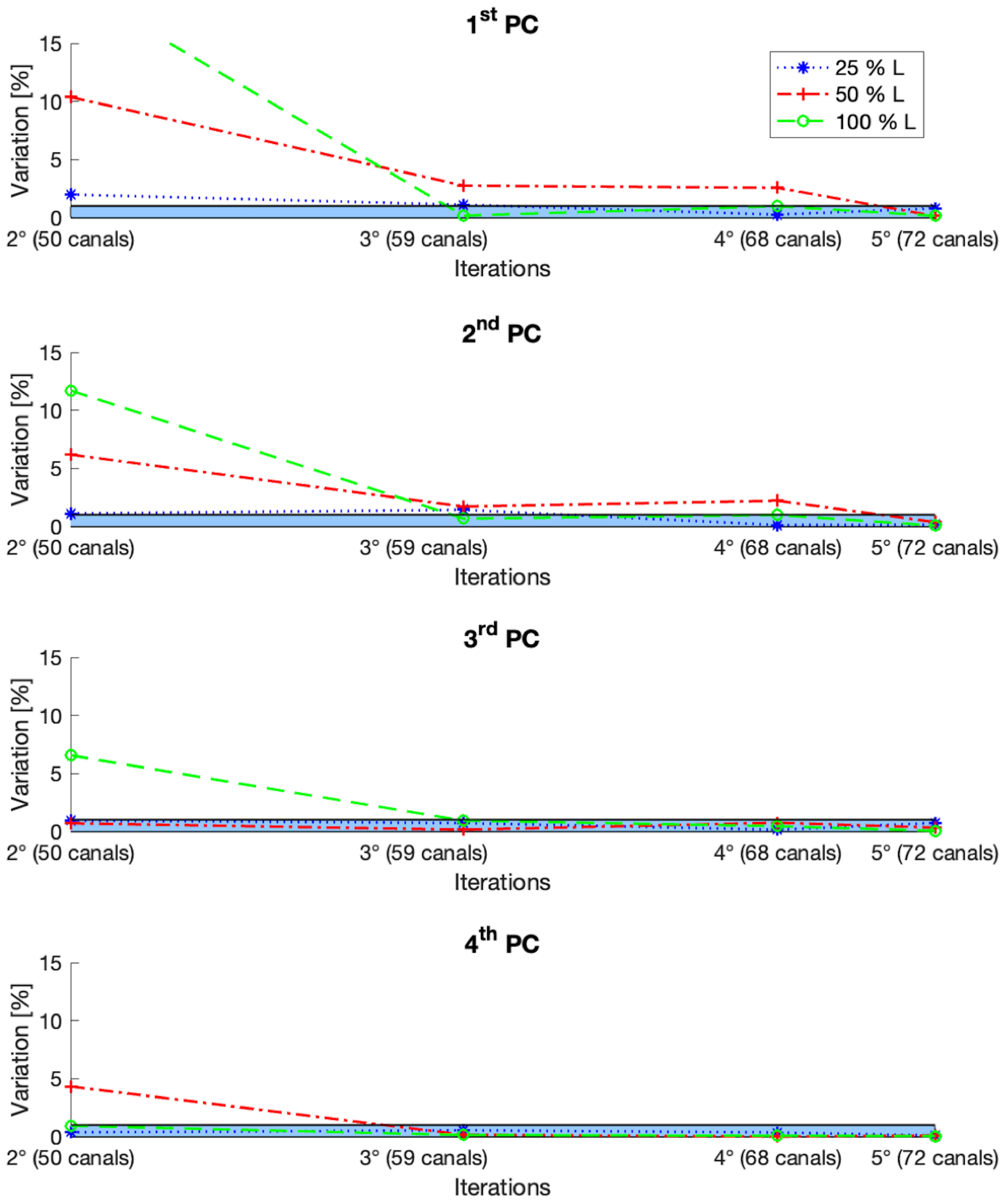

Figure S1.1: variation between the variance explained by the first four PCs for the three different canal segments considered (25%, 50%, and 100% L, with blue dotted line, red dash-dotted line and green dashed line respectively). The first iteration was not plotted. In all of the additional four iterations, the variance explained by the first three PC was compared with the variance at the previous iteration; the variance obtained with the SSMs computed with 50 canals (2° iteration) was compared with the one obtained with 22 canals (1° iteration). The blue band underlines the area where the variation was less than 1%.

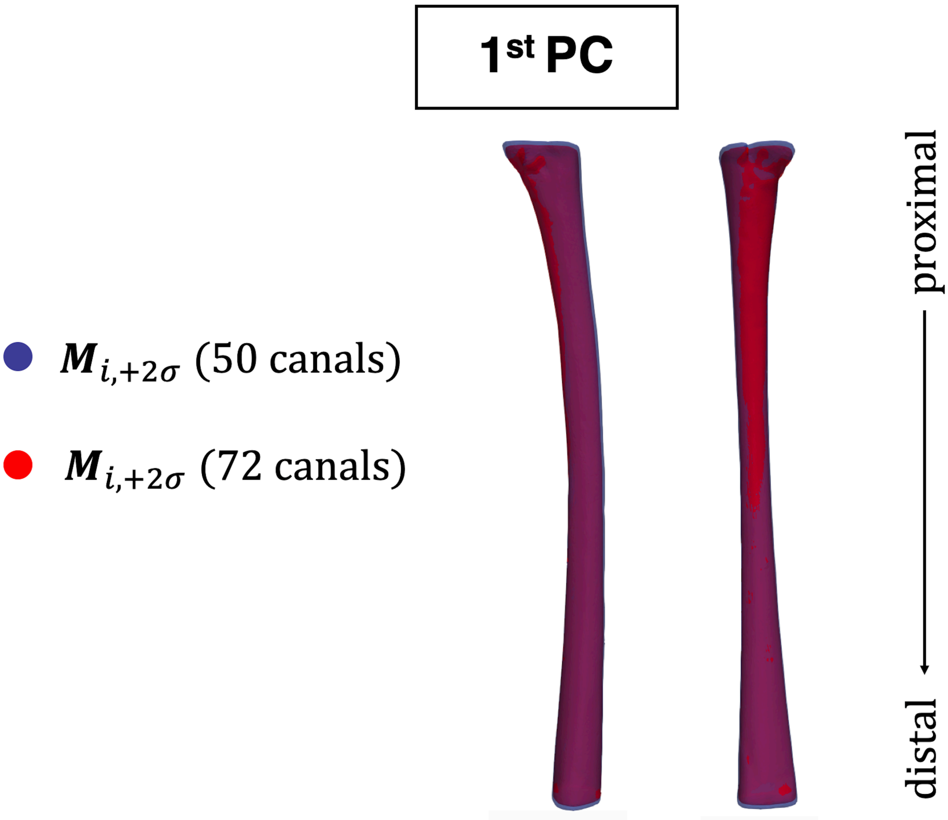

Figure S1.2: a sagittal (on the left) and coronal (on the right) view of the shapes that deviate $+2\sigma$ *deviations from the average shape obtained for the SSMs computed with 50 (in blue) and 72 canals (in red) for the* 1st *PC.*
